# Supplementary material for: A novel pathway of LPS uptake through syndecan-1 leading to pyroptotic cell death
Source: eLife. 2018 Dec 7;7:e37854. doi: 10.7554/eLife.37854 (PMC6286126; doi:10.7554/eLife.37854)
Supplement: Supplementary file 1. [file elife-37854-supp1.docx]

|  | LPS contents (EU/µg) |
| --- | --- |
| Human SCGB3A2 (C1) | 0.00936 |
| Human SCGB3A2 (C2) | 0.00264 |
| Mouse SCGB3A2 (C3) | 13.4 |
| Mouse SCGB3A2 (C4) | 0.0767 |
| Mouse SCGB3A2-3 (C5) | 0.34 |
| Mouse SCGB3A2-4 (C6) | 0.53 |
| GST- Mouse SCGB3A2 (C7) | <0.1 |
| His- Mouse SCGB3A2 (C8) | <0.1 |
