# Supplementary material for: A novel pathway of LPS uptake through syndecan-1 leading to pyroptotic cell death
Source: eLife. 2018 Dec 7;7:e37854. doi: 10.7554/eLife.37854 (PMC6286126; doi:10.7554/eLife.37854)
Supplement: Supplementary file 3. [file elife-37854-supp3.docx]

| Gene symbol | Description | Receptor type | Database ID |
| --- | --- | --- | --- |
| **GYPC** | Glycophorin C (Gerbich blood group) | Single-pass type III membrane  glycoprotein | NM_016815.2 |
| **DPCR1** | Diffuse panbronchiolitis critical region 1 | Single-pass type I membrane protein | NM_080870.2 |
| **SDC1** | Syndecan-1 | Single-pass type I membrane heparin sulfate proteoglycan | NM_001006946.1 |
| **CASC4** | Cancer susceptibility candidate 4 | Single-pass type II membrane protein | NM_177974.1 |
| **TMEM14B** | Transmembrane protein 14B | Multi-pass (4) membrane protein | BC007080.1 |
| **PDPN** | Podoplanin | type I integral membrane glycoprotein | NM_001006624.1 |
| **FXYD7** | FXYD domain containing ion transport regulator 7 | Single-pass membrane protein | NM_022006.1 |
| **CLEC7A** | C-type lectin domain family 7, member A | Small type II membrane glycoprotein receptor | BC013385.1 |
| **SELPLG** | Selectin P ligand | High affinity counter-receptor for P-selectin | NM_003006.2 |
| **FAS** | Tumor necrosis factor receptor superfamily member 6 | Cysteine-rich type I transmembrane receptor | NM_152876.1 |
| **EPHA3** | Ephrin receptor A3 | Receptor protein tyrosine kinase family | PV3359 |
| **P2RY11** | P2Y purinoceptor 11 | G protein coupled receptor, multi-pass membrane protein | BC009877.1 |
| **SMAP1** | Stromal membrane-associated protein 1 | Type II membrane glycoprotein | BC036123.1 |
